# Supplementary material for: Longitudinal Internal Validity of the Quality of Life after Brain Injury: Response Shift and Responsiveness
Source: J Clin Med. 2023 Apr 29;12(9):3197. doi: 10.3390/jcm12093197 (PMC10179561; doi:10.3390/jcm12093197)
Supplement: Supplementary file 1 [file jcm-12-03197-s001.zip › S2_Practical Significance.pdf]

Practical Significance – Probabilities of choosing a particular response category under the corresponding model

Cognition

	Threshold Invariance Model					Residual Invariance Model				
	C0	C1	C2	C3	C4	C0	C1	C2	C3	C4
3 months post TBI										
Concentration	0.04	0.09	0.19	0.41	0.27	0.04	0.09	0.20	0.42	0.26
Expression	0.02	0.06	0.15	0.41	0.36	0.02	0.06	0.15	0.42	0.37
Memory	0.04	0.11	0.23	0.36	0.26	0.04	0.11	0.24	0.36	0.25
Solutions	0.03	0.06	0.15	0.37	0.40	0.02	0.06	0.15	0.38	0.39
Decisions	0.02	0.06	0.16	0.40	0.35	0.02	0.06	0.16	0.40	0.35
Navigate	0.02	0.04	0.12	0.35	0.47	0.02	0.04	0.12	0.35	0.47
Speed	0.03	0.07	0.21	0.40	0.30	0.02	0.07	0.21	0.40	0.29
6 months post TBI										
Concentration	0.03	0.09	0.20	0.44	0.24	0.03	0.09	0.20	0.43	0.25
Expression	0.02	0.05	0.15	0.42	0.36	0.02	0.05	0.15	0.42	0.36
Memory	0.04	0.11	0.24	0.36	0.25	0.04	0.11	0.23	0.36	0.24
Solutions	0.02	0.06	0.15	0.40	0.39	0.02	0.06	0.15	0.38	0.39
Decisions	0.02	0.06	0.16	0.40	0.35	0.02	0.06	0.16	0.40	0.35
Navigate	0.02	0.04	0.12	0.35	0.47	0.02	0.04	0.12	0.36	0.47
Speed	0.02	0.06	0.22	0.42	0.28	0.02	0.07	0.22	0.41	0.29

Note. Response Categories: C0 = “Not at all”; C1 = “Slightly”; C2 = “Moderately”; C3 = “Quite”; C4 = “Very”.

Self

	Loadings Invariance Model					Threshold Invariance Model				
	C0	C1	C2	C3	C4	C0	C1	C2	C3	C4
3 months post TBI										
Energy	0.06	0.14	0.27	0.36	0.18	0.06	0.14	.027	0.36	0.17
Motivation	0.04	0.11	0.25	0.38	0.21	0.04	0.11	0.26	0.39	0.21
Self-Esteem	0.04	0.10	0.22	0.39	0.25	0.04	0.10	0.22	0.39	0.24
Appearance	0.04	0.09	0.23	0.43	0.21	0.04	0.09	0.24	0.43	0.20
Achievements	0.04	0.07	0.16	0.36	0.37	0.04	0.07	0.16	0.37	0.36
Perception	0.03	0.08	0.21	0.44	0.23	0.03	0.08	0.21	0.45	0.23
Future	0.04	0.09	0.22	0.41	0.24	0.04	0.11	0.24	0.38	0.24
6 months post TBI										
Energy	0.06	0.13	0.26	0.36	0.19	0.06	0.13	0.26	0.35	0.19
Motivation	0.04	0.11	0.24	0.38	0.23	0.04	0.11	0.24	0.38	0.24
Self-Esteem	0.04	0.10	0.21	0.38	0.27	0.04	0.10	0.21	0.38	0.27
Appearance	0.04	0.09	0.23	0.43	0.22	0.04	0.09	0.22	0.42	0.23
Achievements	0.04	0.07	0.15	0.36	0.38	0.04	0.07	0.15	0.35	0.39
Perception	0.03	0.08	0.20	0.44	0.25	0.03	0.08	0.20	0.43	0.26
Future	0.04	0.09	0.21	0.41	0.25	0.04	0.09	0.21	0.40	0.26

Note. Response Categories: C0 = “Not at all”; C1 = “Slightly”; C2 = “Moderately”; C3 = “Quite”; C4 = “Very”.

Daily Life & Autonomy

	Threshold Invariance Model					Residual Invariance Model				
	C0	C1	C2	C3	C4	C0	C1	C2	C3	C4
3 months post TBI										
Extent of Ind.	0.03	0.07	0.15	0.38	0.36	0.03	0.07	0.16	0.37	0.37
Out and About	0.05	0.07	0.13	0.29	0.46	0.05	0.06	0.14	0.30	0.45
Domestic Act.	0.05	0.07	0.15	0.32	0.41	0.05	0.08	0.16	0.30	0.42
Finances	0.09	0.07	0.13	0.26	0.45	0.09	0.07	0.13	0.26	0.45
Work/Education	0.10	0.12	0.19	0.32	0.27	0.10	0.12	0.19	0.33	0.26
Social Act.	0.09	0.13	0.22	0.32	0.24	0.08	0.14	0.22	0.31	0.25
Own Life	0.05	0.09	0.18	0.36	0.33	0.04	0.09	0.17	0.36	0.33
6 months post TBI										
Extent of Ind.	0.02	0.06	0.13	0.36	0.42	0.03	0.06	0.127	0.37	0.41
Out and About	0.03	0.05	0.11	0.28	0.52	0.03	0.06	0.11	0.28	0.53
Domestic Act.	0.04	0.06	0.13	0.30	0.47	0.04	0.06	0.12	0.32	0.47
Finances	0.07	0.06	0.12	0.25	0.51	0.07	0.06	0.11	0.25	0.50
Work/Education	0.08	0.10	0.19	0.33	0.31	0.08	0.09	0.19	0.33	0.31
Social Act.	0.07	0.11	0.21	0.33	0.29	0.07	0.10	0.21	0.34	0.29
Own Life	0.03	0.07	0.16	0.37	0.38	0.03	0.07	0.16	0.36	0.38

Note. Response Categories: C0 = “Not at all”; C1 = “Slightly”; C2 = “Moderately”; C3 = “Quite”; C4 = “Very”.

Social Relationships

	Loadings Invariance Model					Threshold Invariance Model				
	C0	C1	C2	C3	C4	C0	C1	C2	C3	C4
3 months post TBI										
Affect	0.00	0.02	0.10	0.42	0.46	0.00	0.02	0.10	0.42	0.46
Family	0.02	0.05	0.12	0.32	0.49	0.03	0.05	0.11	0.33	0.49
Friends	0.02	0.04	0.14	0.38	0.43	0.02	0.04	0.14	0.38	0.43
Partner	0.07	0.07	0.13	0.27	0.47	0.07	0.07	0.12	0.28	0.47
Sex	0.14	0.10	0.20	0.29	0.27	0.14	0.10	0.21	0.28	0.27
Attitudes	0.02	0.06	0.17	0.43	0.33	0.02	0.05	0.18	0.43	0.33
6 months post TBI										
Affect	0.00	0.02	0.10	0.40	0.47	0.00	0.03	0.10	0.40	0.47
Family	0.03	0.05	0.12	0.32	0.50	0.02	0.05	0.12	0.31	0.50
Friends	0.02	0.04	0.14	0.37	0.43	0.02	0.05	0.14	0.36	0.43
Partner	0.07	0.06	0.13	0.27	0.48	0.06	0.06	0.14	0.27	0.47
Sex	0.13	0.10	0.21	0.30	0.27	0.13	0.09	0.20	0.31	0.27
Attitudes	0.02	0.05	0.18	0.43	0.32	0.02	0.06	0.17	0.44	0.32

Note. Response Categories: C0 = “Not at all”; C1 = “Slightly”; C2 = “Moderately”; C3 = “Quite”; C4 = “Very”.

Emotions

	Threshold Invariance Model					Residual Invariance Model				
	C0	C1	C2	C3	C4	C0	C1	C2	C3	C4
3 months post TBI										
Lonely	0.03	0.09	0.13	0.24	0.50	0.03	0.10	0.13	0.25	0.50
Bored	0.05	0.12	0.16	0.28	0.39	0.05	0.12	0.16	0.29	0.38
Anxious	0.05	0.11	0.16	0.25	0.43	0.05	0.11	0.16	0.25	0.43
Sad	0.06	0.13	0.16	0.24	0.40	0.06	0.13	0.16	0.25	0.39
Angry	0.05	0.10	0.12	0.24	0.48	0.06	0.10	0.12	0.24	0.47
6 months post TBI										
Lonely	0.03	0.09	0.13	0.25	0.51	0.03	0.09	0.13	0.24	0.51
Bored	0.04	0.12	0.16	0.30	0.39	0.05	0.12	0.16	0.29	0.39
Anxious	0.05	0.11	0.16	0.25	0.44	0.05	0.11	0.16	0.25	0.44
Sad	0.06	0.13	0.16	0.25	0.40	0.06	0.13	0.16	0.25	0.41
Angry	0.06	0.10	0.12	0.24	0.48	0.05	0.10	0.12	0.24	0.49

Note. Response Categories: C0 = “Not at all”; C1 = “Slightly”; C2 = “Moderately”; C3 = “Quite”; C4 = “Very”.

Physical Problems

	Loadings Invariance Model					Threshold Invariance Model				
	C0	C1	C2	C3	C4	C0	C1	C2	C3	C4
3 months post TBI										
Slow	0.06	0.13	0.14	0.25	0.43	0.06	0.13	0.14	0.25	0.42
Injuries	0.12	0.14	0.14	0.20	0.41	0.12	0.15	0.14	0.19	0.41
Pain	0.06	0.14	0.16	0.28	0.36	0.06	0.13	0.16	0.29	0.36
See/Hear	0.06	0.11	0.13	0.21	0.50	0.06	0.11	0.12	0.20	0.52
Brain	0.08	0.13	0.17	0.27	0.35	0.08	0.12	0.17	0.28	0.35
6 months post TBI										
Slow	0.05	0.11	0.13	0.24	0.47	0.04	0.11	0.13	0.25	0.47
Injuries	0.10	0.13	0.13	0.20	0.45	0.10	0.12	0.12	0.20	0.46
Pain	0.06	0.12	0.15	0.26	0.41	0.06	0.12	0.15	0.26	0.41
See/Hear	0.05	0.10	0.12	0.20	0.54	0.05	0.10	0.12	0.21	0.52
Brain	0.06	0.11	0.16	0.28	0.39	0.06	0.12	0.16	0.27	0.40

Note. Response Categories: C0 = “Not at all”; C1 = “Slightly”; C2 = “Moderately”; C3 = “Quite”; C4 = “Very”.

Practical Significance – Discrepancies between invariance models

Cognition

Differences Threshold vs. Residual Model

	Not at all	Slightly	Moderately	Quite	Very
3 months post TBI					
Concentration	0	0	0	0.01	0.01
Expression	0	0	0	0	0
Memory	0	0	0	0	0.01
Solutions	0	0	0	0.01	0.01
Decisions	0	0	0	0.01	0.01
Navigate	0	0	0	0	0
Speed	0	0	0	0.01	0.01
6 months post TBI					
Concentration	0	0	0	0.01	0.01
Expression	0	0	0	0	0
Memory	0	0	0	0	0.01
Solutions	0	0	0	0.01	0.01
Decisions	0	0	0	0.01	0.01
Navigate	0	0	0	0	0
Speed	0	0	0	0.01	0.01

Note. The values represent the differences between the probabilities of choosing a particular response category for the models with different constraints. Multiplying by 100 gives percentages.

Self

Threshold vs. Residual Model

	Not at all	Slightly	Moderately	Quite	Very
3 months post TBI					
Energy	0	0	0	0	0.01
Motivation	0	0	0	0	0.01
Self-Esteem	0	0	0	0.01	0.01
Appearance	0	0	0	0	0.01
Achievements	0	0	0	0.01	0.01
Self-Perception	0	0	0	0.01	0.01
Future	0	0	0	0.01	0.01
6 months post TBI					
Energy	0	0	0	0	0.01
Motivation	0	0	0	0	0.01
Self-Esteem	0	0	0	0.01	0.01
Appearance	0	0	0	0	0.01
Achievements	0	0	0	0.01	0.01
Self-Perception	0	0	0	0.01	0.01
Future	0	0	0	0.01	0.01

Note. The values represent the differences between the probabilities of choosing a particular response category for the models with different constraints. Multiplying by 100 gives percentages.

Daily Life & Autonomy

Threshold vs. Loading Model

	Not at all	Slightly	Moderately	Quite	Very
			3 months post TBI		
Extent of Ind.	0	0	0.01	0.01	0.01
Out and About	0	0	0	0.01	0.01
Domestic Act.	0	0	0.01	0.02	0
Finances	0	0	0	0	0
Work/Education	0	0.01	0	0	0
Social Act.	0	0.01	0.01	0.01	0
Own Life	0	0	0	0	0
			6 months post TBI		
Extent of Ind.	0	0	0.01	0.01	0.01
Out and About	0	0	0	0.01	0.01
Domestic Act.	0	0.01	0.01	0.02	0.01
Finances	0.01	0	0	0	0.01
Work/Education	0	0.01	0	0	0
Social Act.	0	0.01	0	0.01	0
Own Life	0	0	0	0	0

Note. The values represent the differences between the probabilities of choosing a particular response category for the models with different constraints. Multiplying by 100 gives percentages.

Social

Differences Loading vs. Threshold Model

	Not at all	Slightly	Moderately	Quite	Very
			3-months post TBI		
Affect	0	0	0	0	0
Family	0	0	0.01	0.01	0.01
Friends	0	0	0	0.01	0
Partner	0	0	0.01	0	0
Sex	0	0	0	0.01	0
Attitudes	0	0	0	0.01	0
			6-months post TBI		
Affect	0	0	0	0	0
Family	0.01	0	0.01	0.01	0
Friends	0	0.01	0	0.01	0
Partner	0.01	0.01	0.01	0	0
Sex	0	0.01	0	0.01	0
Attitudes	0	0.01	0.01	0.01	0

Note. The values represent the differences between the probabilities of choosing a particular response category for the models with different constraints. Multiplying by 100 gives percentages.

Emotions

Differences Threshold vs. Residual Model

	Not at all	Slightly	Moderately	Quite	Very
3 months post TBI					
Lonely	0	0	0	0	0
Bored	0	0	0	0.01	0
Anxious	0	0	0	0	0
Sad	0	0	0	0	0
Angry	0	0	0	0	0
6 months post TBI					
Lonely	0	0	0	0	0
Bored	0	0	0	0.01	0
Anxious	0	0	0	0	0
Sad	0	0	0	0	0
Angry	0	0	0	0	0

Note. The values represent the differences between the probabilities of choosing a particular response category for the models with different constraints. Multiplying by 100 gives percentages.

Physical Problems

Differences Loadings vs. Threshold

	Not at all	Slightly	Moderately	Quite	Very
3 months post TBI					
Slow	0	0	0	0	0
Injuries	0	0.01	0.01	0.01	0
Pain	0	0	0	0.01	0
Seeing Hearing	0	0	0.01	0.01	0.02
Brain	0	0.01	0	0.01	0
6 months post TBI					
Slow	0.01	0	0	0.01	0
Injuries	0.01	0.01	0.01	0.01	0
Pain	0	0	0	0.01	0
Seeing Hearing	0	0	0.01	0.01	0.02
Brain	0	0.01	0	0.01	0

Note. The values represent the differences between the probabilities of choosing a particular response category for the models with different constraints. Multiplying by 100 gives percentages.
